# Aplasia in Chronic Phase CML Post-TKI Therapy: A Management Dilemma

**DOI:** 10.1155/2019/4861673

**Published:** 2019-09-22

**Authors:** Jeremy L. Ramdial, Luis E. Aguirre, Robert A. Ali, Ronan Swords, Mark Goodman

**Affiliations:** ^1^Department of Hematology/Oncology, Sylvester Comprehensive Cancer Center, University of Miami Miller School of Medicine, Miami, FL, USA; ^2^Department of Internal Medicine, University of Miami Miller School of Medicine, Miami, FL, USA

## Abstract

Transient cytopenias and bone marrow hypoplasia commonly occur during treatment of CML with TKIs (tyrosine kinase inhibitors). This is usually related to the eradication of CML clones that initially compose the majority of hematopoietic cells in the bone marrow at the time of diagnosis. With continuation of effective therapy, normal blood counts return as normal hematopoiesis is restored and CML clones are reduced. Though rare and more unusual than myelodysplastic syndrome (MDS), isolated instances of persistent marrow aplasia have been documented with chronic use of TKIs. We describe two such instances of chronic phase CML where no significant reduction of CML clones was achieved following treatment with TKIs, but bone marrow aplasia occurred resulting in persistent dysfunctional hematopoiesis. Due to prolonged aplasia/hypoplasia, such patients are no longer amenable to TKI treatment. CML progression to accelerated or blast phase in that setting would likely be fatal.

## 1. Background

TKI-induced marrow aplasia is a rarely described and poorly characterized entity. Known accounts of this interesting phenomenon remain scarce throughout the literature and highlight how challenging their management can be. The consequences can be devastating for afflicted patients. An extensive review of the literature using Pubmed and Trip database with the words “Aplasia,” “Chronic phase,” “CML,” and “TKI/Tyrosine kinase inhibitors” revealed only a handful of published case descriptions. It is with this in mind that we illustrate two local cases of chronic phase CML complicated with aplasia post-TKI therapy. To the extent of our knowledge, these cases represent the first reported instances in which patients developed this complication without having exhibited any meaningful response to therapy. We hope that public awareness of these 2 cases will contribute to expand current knowledge of this phenomenon and ultimately help bring further insight into this process.

## 2. Case Presentation

### 2.1. Case 1

A 64-year-old male with no comorbidities presented to our institution complaining of chronic fatigue and malaise in the setting of underlying hematologic abnormalities. On presentation, preliminary studies were significant for profound leukocytosis of 323 × 10^3^/*μ*L and platelet count of 428 × 10^3^/*μ*L with 5% blasts and 10% basophils in peripheral blood. CT scan of the abdomen revealed massive splenomegaly with an enlarged spleen measuring 24.5 cm in its largest craniocaudal dimensions. Further workup was consistent with chronic phase Ph (+) CML with a breakpoint at p210 and JAK2 negative status. The Sokal index was 2.1 and consistent with high risk disease (which translated to two-year survival rate of 65% and median survival estimated at 2.5 years). The EUTOS score obtained prior to starting TKI therapy was 132 and compatible with high-risk disease (5-year progression-free survival of 82% and complete cytogenic response (CCyR) of 66% at 18 months with a 31% likelihood of not achieving CCyR). Bone marrow FISH showed 81% BCR-ABL transcripts for which he was started on imatinib, achieving hematological response by the 3rd week of treatment and suboptimal cytological response by month 7. The clinical course was complicated by therapy-related pancytopenia/aplastic anemia on month 8. Consequently, imatinib was held for 2 months which allowed his counts to fully recover by month 11.

Routine studies done at follow-up showed marked leukocytosis. Repeat BM biopsy was consistent with chronic phase CML with 5% blasts. Cytogenetics showed t(9; 22) and 3q inversion with 95% BCR-ABL transcripts, and the patient was started on dasatinib. By the 12th month of therapy, decision was made to treat him as accelerated phase due to clonal evolution, with consideration for potential allogeneic HSCT once response to dasatinib had been achieved. He was maintained on TKI therapy until month 15 when repeat FISH from peripheral blood showed 79% BCR-ABL transcripts with underlying severe pancytopenia. Dasatinib was held until counts recovered on Neupogen and biweekly transfusion of blood products and restarted subsequently at 80 mg.

Bone marrow biopsy on month 16 showed marked hypocellularity secondary to panmyeloid hypoplasia. Bone marrow cytogenetics demonstrated t(9; 22), inv(3) (q21q26), and 36% BCR-ABL transcripts. Dasatinib was reduced to 70 mg with additional reduction to 50 mg PO daily by month 17 due to persistent pancytopenia. Peripheral blood FISH on month 19 showed 70% BCR-ABL transcripts which led to discontinuation of Dasatinib and institution of omacetaxine by month 21.

On month 25, the patient was admitted to undergo allogeneic MUD/BMT. He was conditioned with Flu/Bu/ATG and received stem cell infusion with successful engrafting on day +15. His post-transplant course was marked by a number of complications including CMV viremia, an episode of seizures attributed to posterior reversible encephalopathy (PRES) and development of myeloid sarcoma discovered incidentally on imaging as a paraspinal mass. BM biopsy at follow-up showed increased percentage of blasts, and he was started on ponatinib 45 mg daily.

### 2.2. Case 2

A 50-year-old male presented to our institution for evaluation of persistent leukocytosis of 26 × 10^3^/*μ*L, newfound thrombocytosis with platelet count of 1042 × 10^3^/*μ*L, and markedly elevated LDH. He had originally noted symptoms of progressive fatigue, weight loss, and general malaise over a period of 6 months. On presentation, the preliminary studies also showed 2% blasts and 5% basophils in peripheral blood. CT scan of the abdomen revealed splenomegaly with an enlarged spleen measuring 16 cm in its largest dimensions. Bone marrow biopsy and additional workup were consistent with chronic phase Ph (+) CML with 137.2% BCR-ABL transcripts, JAK2 negative status, and a breakpoint at p210. The Sokal index was 1.4 and consistent with high-risk disease. The EUTOS score obtained prior to initiation of TKI therapy was 63 and compatible with low-risk disease (5-year progression-free survival of 90% with probability of achieving complete cytogenic response (CCyR) at 18 months of 86% and likelihood of not achieving CCyR of 18%.

Two months after his original presentation, he was started on imatinib therapy at 400 mg QD. Nevertheless, and due to unforeseen circumstances, he was unable to get treatment during months 3 to 4. He returned to clinic on month 5. Imatinib was restarted at a lower dose of 200 mg due to reported GI toxicity. The patient endorsed compliance from months 5 to 10 resulting in hematological response. On month 11, repeat assessment of peripheral blood showed 190% BCR-ABL transcripts. The patient was lost again to follow-up until month 15.

Repeat analysis prior to restarting imatinib showed 190.9% BCR-ABL transcripts. After 3 months on TKI therapy, BCR-ABL transcripts had decreased to 90%. On month 19, he was switched to dasantinib due to suboptimal response. By the end of month 24, treatment was switched to nilotinib due to interim development of intolerance to dasatinib.

On month 26, a repeat biopsy while on nilotinib showed chronic phase CML with a hypocellular marrow with trilineage hypoplasia. Cytogenetic analysis showed trisomy 8 and t(9 : 22), a 46 XY karyotype and negative ABL1 kinase domain mutation. After 4 months of treatment, his counts normalized with the exception of persistent mild thrombocytopenia. Nilotinib dose was reduced from 400 mg to 200 mg due to development of worsening cytopenias, but his counts continued to deteriorate over time, prompting hospital admission on month 33 with flow cytometry showing 7% blasts. He was discharged after transfusion of platelets but was readmitted a few weeks later for rectal bleeding in the setting of pancytopenia. Repeat bone marrow biopsy showed a hypocellular marrow with stromal edema and no significant hematopoiesis consistent with therapy effect. Blasts were not increased. Given these results, he continued on nilotinib for one more month.

A repeat biopsy conducted on month 34 showed variable cellularity with left shifted granulopoiesis, focal megakaryocytic hyperplasia, dysmegakaripoiesis, stromal fibrosis, and plasmacytosis with 5% blasts. BCR-ABL transcripts were 95%. TKI therapy was promptly discontinued on month 35 after concluding that persistent cytopenias were a consequence of nilotinib treatment rather than disease progression.

Repeat marrow biopsy on month 37 showed a normocellular marrow with left-shifted myelopoiesis and no blasts. Cytogenetics confirmed 42% BCR-ABL transcripts.

Over the next months, the patient developed therapy-related aplastic anemia with severe pancytopenia. His counts eventually improved with eltrombopag while undergoing studies for allogeneic HSCT. Follow-up bone marrow biopsy two months after eltrombopag therapy showed a hypocellular marrow with 10–15% lymphoblasts suggestive of lymphoid blast phase CML. Studies showed persistent BCR-ABL transcripts (8% by PCR and 22% by FISH with no other cytogenetic abnormalities). He stopped responding to eltrombopag and became markedly thrombocytopenic. His condition declined precipitously culminating with a hemorrhagic stroke on month 45 and profuse hemorrhage leading to death.

## 3. Discussion

In CML, cellular proliferation and clonal expansion are driven by constitutively active BCR-ABL tyrosine kinase [1]. TKIs such as imatinib, nilotinib, and dasatanib have become the mainstay of therapy for Ph + CML by exerting selective inhibition of the BCR-ABL fusion protein through competitive binding at the ATP-binding site [2]. Continuous use of these drugs has been shown to achieve durable response in patients, and therefore awareness of their associated complications and methods to treat them becomes important.

Cytopenias are known to occur during treatment of CML. In 2016, Poudyal et al. described a patient who developed pure red cell aplasia (PRCA) from treatment with imatinib and nilotinib [3]. Sumi, Khan, Estephan, Prodduturi, Lokeshwar, and Song have each described patients developing aplasia after exposure to single BCR-ABL TKI (Table 1) [4–9].

**Figure 1:**
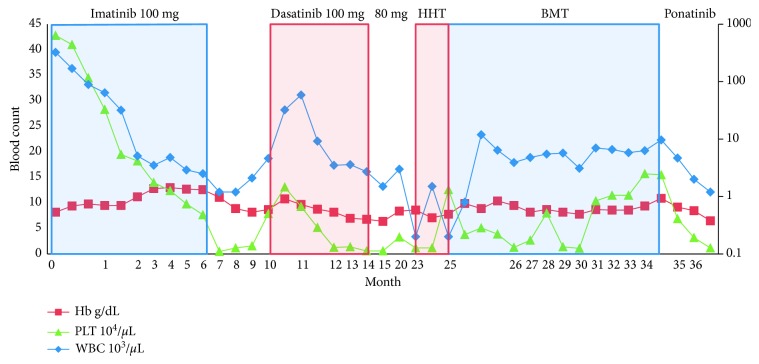
Hematologic parameters in patients with CML treated with imatinib, dasatinib, subsequent BMT, and ponatinib following relapse. White blood cells (WBC), hemoglobin (Hb), and platelets (PLTs) are depicted over a 36-month period until the last day of patient follow-up. The graph is divided into seven frames: (1) initial response to imatinib 100 mg followed by (2) discontinuation due to pancytopenia. (3) Treatment was switched to dasatinib 100 mg followed by (5) subsequent reductions in dosing in the setting of persistent pancytopenia. (6) The patient was switched to omacetaxine due to proven panmyeloid hypoplasia leading to (8) BMT. (9) The patient was started on ponatinib due to relapsed disease.

**Figure 2:**
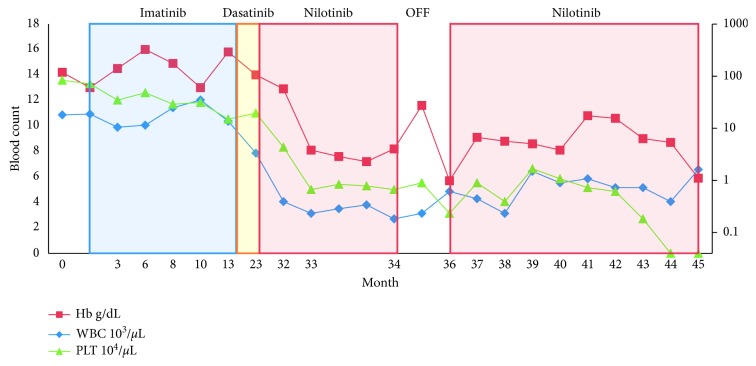
Hematologic parameters in patients with CML treated with imatinib, dasatinib, and subsequent nilotinib. White blood cells (WBC), hemoglobin (Hb), and platelets (PLTs) are depicted over a 45-month period until patient's death. The graph is divided into five frames: (1) initiation of imatinib and lack of adequate response due to noncompliance. Subsequent dose adjustments and continuation of treatment until month 18. (2) Initiation of dasatinib following discontinuation of imatinib due to lack of proper response. (3) Treatment was switched to nilotinib on month 23 due to persistent cytopenias and intolerable side effects. Bone marrow biopsy on month 25 revealed trilineage hypoplasia. (5) Discontinuation of nilotinib on month 34 due to persistent pancytopenia and marrow fibrosis attributed to TKI. (6) Decision to resume nilotinib with plan for urgent BMT frustrated by complications leading to death of the patient on month 45.

Figures 1 and 2 show the trend of the hematologic parameters of patients 1 and 2 over their course of treatment with TKIs. In general, there are three reasons for therapy-related cytopenic events in CML. First, there is transient bone marrow hypoplasia at the onset of therapy related to reduction in CML clones during a time where normal hematopoiesis has not yet been restored. In this case, bone marrow hypoplasia/aplasia is usually accompanied by marked reduction of CML clones as judged by cytogenetics, FISH, and PCR for BCR-ABL. This was not the case in our two patients as aplasia was not associated with significant reduction in CML markers. Second, during therapy, some patients may develop new cytogenetic abnormalities (most commonly trisomy 8), suggesting development of myelodysplastic syndrome (MDS). In these instances, there is no marrow aplasia either. Third, some patients may develop into a blast phase and present with cytopenias. In these cases, marrows are also not aplastic.

While the patients described in our case series also developed aplasia, they never achieved cytological remission, in contrary to all other published reports. As such, both of our patients did poorly. It is important to notice, however, that both of them were identified as high-risk patients according to their Sokal scores (2.1 and 1.4 for patients 1 and 2, respectively). Indexes above 1.2 are considered high risk which translates to a two-year survival rate of 65% and estimated median survival of 2.5 years. Based on the Sokal score alone, these data mirror the well-known fact that patients with high risk disease are less prone to achieve cytological remission. High-risk patients who develop recurrent cytopenias on treatment that require dosing adjustments and sequential exposure to different TKIs may be more prone to develop aplasia.

There was less of an association between EUTOS scores (high risk for patient 1 and low risk for patient 2) and risk of developing marrow aplasia after TKI treatment. Both of them failed to achieve cytological remission. The reason for this disparity between our patients' Sokal and EUTOS risk stratification lies in fact that the data analyzed to develop these prognostic models were obtained from derivation studies conducted in radical different treatment eras [10, 11]. Given that the original study by Sokal and colleagues was conducted in 1984 in the pre-imatinib era and that treatment with TKIs has radically changed the prognosis of patients with CML since their introduction, the Sokal score may no longer be a strong predictor of the outcome in these patients according to a number of experts [11]. The EUTOS score may be a better predictor of outcome in CML after treatment with TKIs, as validated by Hoffman, Tribelli, Breccia, Uz, and Yahng, respectively [11–17], but there are also at least 3 studies (Marin, Jabbour, and Yamamoto) that question its validity [18–20]. This issue is still a matter of active debate.

## 4. Conclusion

Small molecule tyrosine kinase inhibitors have revolutionized the treatment of chronic myeloid leukemia. Transient myelosuppression is a common adverse event seen in patients taking TKIs; however, severe and prolonged pancytopenia together with BM aplasia secondary to TKI therapy is uncommon and only found in a handful of case reports. High-risk patients with Sokal scores greater than 1.2 who develop recurrent cytopenias on treatment that requires dosing adjustments and sequential exposure to different TKIs may be more prone to develop marrow aplasia.

Our cases describe the possible outcomes in these patients, with one of them able to undergo transplant by 24 months and the other unfortunately passing by month 45. Early recognition of this phenomenon followed by prompt transplant referral may prove to be crucial in order to prevent catastrophic consequences.

## Figures and Tables

**Table 1:** Description of reported cases of TKI-related Bone Marrow Hypoplasia/Aplasia.

	Demographics	Phase of CML	TKI therapy	Timeline for cytopenias	Bone marrow biopsy	Clinical course
Lokeshwar et al. [4]	46 yo, male	Chronic phase CML	Imatinib 400 mg/d. Prior treatments: busulfan, IFN*α* + low-dose Ara-C, hydroxyurea.	Pancytopenia at week 6.	D56: severely hypoplastic (5%) and fatty; no significant fibrosis. Blast cells not appreciable.	Admitted for febrile neutropenia secondary to gastroenteritis and *Escherichia coli* bacteremia. She clinically declined with the development of bilateral pulmonary infiltrates. The patient passed, with postmortem confirming pulmonary mucormycosis.
Sumi et al. [5]	73 yo, female	Chronic phase CML	Imaitinib 400 mg/d. Prior treatments: IFN*α*, hydroxyurea, busulfan.	Pancytopenia at week 17 GIV neutropenia at day 35 GIII thrombocytopenia at day 122.	D87: severe hypocellularity.	Supportive care: transfusion PRBC, platelet; filgrastim.
Khan et al. [6]	51 yo, male	Chronic phase CML	Imatinib 400 mg daily initially, then dose reduced to 300 mg daily, then 200 mg daily for thrombocytopenia.	Anemia and thrombocytopenia at week 13, progressing to pancytopenia by week 19.	D126: slightly depressed cellularity.	Deceased: pulmonary tuberculosis, liver failure, worsening pancytopenia. Passed away despite supportive care.
Song et al. [7]	77 yo, male	Chronic phase CML	Imatinib 400 mg PO daily, dose reduced to 300 mg secondary to patient intolerance, then eventually discontinued. Nilotinib 400 mg po BID.	CCR after 9 months of imatinib therapy. No evidence of pancytopenia. Pancytopenia at week 8 of nilotinib therapy.	D252 (imatinib): CCR D56 (nilotinib): <5% normal cellularity. Fatty tissue without myelofibrosis or adipocyte deposition. No chromosomal abnormalities.	Four months after discontinuation of nilotinib, the patient remained in CCR, in the absence of further CML-specific therapy. Hematologic parameters did not recover by this time.
Poudyal et al. [3]	35 yo, male	Chronic phase CML	Imatinib 400 mg po daily. Rechallenged with 300 mg po daily after recovery of PRCA. Eventually switched to Nilotinib 400 mg po BID, then dose-reduced to 200 mg po BID secondary to recurrence of PRCA.	Anemia with reticulopenia at week 16. Anemia recurred with rechallenge of imatinib 300 mg, at week 8. Anemia recurred with nilotinib 400 mg BID, at day 40.	Three BMBx performed, each prompted by anemia while on TKI. Each BMBx demonstrated PRCA.	PRCA developed with initial dose of imatinib 400 mg daily. Discontinuation of TKI and initiation of prednisone 1 mg/kg resulted in recovery of marrow by D21. Rechallenge with imatinib 300 mg daily also resulted in recurrence of PRCA but was steroid refractory. Oral cyclosporine facilitated marrow recovery. Nilotinib 400 mg po BID was initiated, but PRCA recurred. Nilotinib was held and cyclosporine continued, with recovery of counts. Nilotinib was resumed at 200 mg BID, with continuous cyclosporine. There were no further declines in hemoglobin.
Estephan et al. [8]	53 yo, female	Chronic phase CML	Nilotinib 300 mg po BID. Discontinued secondary to pancytopenia. Switched to Dasatinib 50 mg daily once, cytopenias recovered.	Pancytopenia at week 10.	D70: hypocellular (<5%) with decreased trilineage hematopoiesis (panhypoplasia) and no residual CML. D140: mormocellular marrow (40%) and trilineage hematopoiesis. No left shift in maturation or increased blasts. Cyteogenetics 46XX.	Romiplostim initiated with development of pancytopenia. By 3 months, intervals between transfusions lengthened. By 5 months, repeat BMBx demonstrated recovery. Dasatinib started with good cytogenetic response.
Prodduturi et al. [9]	49 yo, male	Chronic phase CML	Imatinib 400 mg po daily. Therapy switched as milestones not achieved at 7 months. Dasatinib 100 mg po daily. Discontinued due to anaphylactic reaction. Nilotinib 400 mg po BID, then daily, then 200 mg daily. Dose reduced and eventually discontinued secondary to cytopenias.	Pancytopenia with nilotinib 400 mg BID at month 6, but with CCR. Pancytopenia recurred after 1 month of dose-reduced nilotinib 200 mg daily.	D168 (nilotinib 400 mg BID): 5–10% cellularity; CCR D364 (nilotinib 400 mg BID): 5% cellularity; CCR D56 (discontinuation of nilotinib): 40% cellularity, Ph^+^ in 13/20 metaphases D30 (Nilotinib 200 mg daily): <5% cellularity; Ph^+^ in 12/20 metaphases.	Milestones not achieved after 7 months of Imatinib 400 mg daily. Anaphylaxis to Dasatinib 100 mg precluded further use. Initially started on nilotinib 400 mg po BID which was decreased to daily secondary to cytopenias. BMBx showed progression, and dose was escalated to twice daily. BMBx at 6 months, then after further 7 months, it showed 5% cellularity but CCR. Marrow recovered following discontinuation of nilotinib but also with disease progression. Thereafter, resumption of nilotinib at 200 mg daily resulted in pancytopenia after 1 month, with persistent disease. All CML-directed therapy discontinued with recovery of peripheral counts.

TKI, tyrosine kinase inhibitor; IFN*α*, interferon alfa; GIII, grade III; GIV, grade IV; CCR, complete cytogenetic remission; CHR, complete hematologic response; PRCA, pure red cell aplasia; BMBx, bone marrow biopsy.
